# Comparative Anatomy of the Dentate Mossy Cells in Nonhuman Primates: Their Spatial Distributions and Axonal Projections Compared With Mouse Mossy Cells

**DOI:** 10.1523/ENEURO.0151-24.2024

**Published:** 2024-05-10

**Authors:** Minseok Jeong, Jinyoung Won, Kyung Seob Lim, Chang-Yeop Jeon, Youngshik Choe, Jin-Hyeok Jang, Chang Man Ha, Jong Hyuk Yoon, Yongjeon Lee, Yong-Seok Oh

**Affiliations:** ^1^Department of Brain Sciences, Daegu-Gyeongbuk Institute of Science and Technology (DGIST), Daegu 42988, Republic of Korea; ^2^National Primate Research Center, Korea Research Institute of Bioscience and Biotechnology (KRIBB), Cheongju 28116, Republic of Korea; ^3^Futuristic Animal Resource and Research Center, Korea Research Institute of Bioscience and Biotechnology (KRIBB), Cheongju 28116, Republic of Korea; ^4^Developmental Disorders & Rare Diseases Research Group, Korea Brain Research Institute (KBRI), Daegu 41062, Republic of Korea; ^5^Research Division and Brain Research Core Facilities, Korea Brain Research Institute (KBRI), Daegu 41062, Republic of Korea; ^6^Neurodegenerative Diseases Research Group, Korea Brain Research Institute (KBRI), Daegu 41062, Republic of Korea; ^7^Department of Functional Genomics, KRIBB School of Bioscience, University of Science and Technology (UST), Daejeon 34113, Republic of Korea; ^8^Emotion, Cognition & Behavior Research Group, Korea Brain Research Institute (KBRI), Daegu 41062, Republic of Korea

**Keywords:** associational projections, commissural projections, dentate gyrus, heterogeneity, hippocampus, mossy cells

## Abstract

Glutamatergic mossy cells (MCs) mediate associational and commissural connectivity, exhibiting significant heterogeneity along the septotemporal axis of the mouse dentate gyrus (DG). However, it remains unclear whether the neuronal features of MCs are conserved across mammals. This study compares the neuroanatomy of MCs in the DG of mice and monkeys. The MC marker, calretinin, distinguishes two subpopulations: septal and temporal. Dual-colored fluorescence labeling is utilized to compare the axonal projection patterns of these subpopulations. In both mice and monkeys, septal and temporal MCs project axons across the longitudinal axis of the ipsilateral DG, indicating conserved associational projections. However, unlike in mice, no MC subpopulations in monkeys make commissural projections to the contralateral DG. In monkeys, temporal MCs send associational fibers exclusively to the inner molecular layer, while septal MCs give rise to wide axonal projections spanning multiple molecular layers, akin to equivalent MC subpopulations in mice. Despite conserved septotemporal heterogeneity, interspecies differences are observed in the topological organization of septal MCs, particularly in the relative axonal density in each molecular layer along the septotemporal axis of the DG. In summary, this comparative analysis sheds light on both conserved and divergent features of MCs in the DG of mice and monkeys. These findings have implications for understanding functional differentiation along the septotemporal axis of the DG and contribute to our knowledge of the anatomical evolution of the DG circuit in mammals.

## Significance Statement

This study investigates glutamatergic mossy cells (MCs) in the dentate gyrus (DG) of mice and monkeys, revealing both conserved and species-specific features. While associational projections are consistent across species, monkeys lack the commissural projections of MCs seen in mice. Additionally, the topological organization of septal MC axons differs, particularly in relative axonal density along the septotemporal axis. These findings provide insights into the anatomical evolution of the DG circuit in mammals, shedding light on potential functional distinctions. The study enhances our understanding of neural circuitry, offering a platform for further exploration into the intricate relationship between structure and function in the mammalian brain.

## Introduction

The hippocampus plays critical roles in both cognitive and affective behaviors, and its dysfunctions are associated with a wide range of brain disorders (Fanselow and Dong, 2010; Strange et al., 2014). The long, curved structure of the hippocampus extends along the dorsoventral axis in rodents and the anterior-to-posterior axis in primates (Poppenk et al., 2013). Based on the traditional dichotomous view, the septal (dorsal in rodents; posterior in primates) hippocampus has been associated with cognitive abilities, such as spatial and episodic memory, while the temporal (ventral in rodents; anterior in primates) side is more with anxiety-related behaviors (Fanselow and Dong, 2010). Septotemporal differentiation of the hippocampal functions is causally linked to spatially patterned within-cell-type heterogeneity as well as to afferent and efferent connectivity within corticolimbic structures (Strange et al., 2014; Cembrowski and Spruston, 2019).

Glutamatergic mossy cells (MCs) mediate associational and commissural connectivity in the bilateral dentate gyrus (DG; Amaral, 1978; Scharfman, 2016). In the context of the DG circuitry, MCs receive dense excitatory inputs from local granule cells (GCs) through synaptic connections on their thorny excrescences (Scharfman and Myers, 2013). The excitatory outputs of MCs have the distinct property of directly activating GCs (Ribak et al., 1985; Buckmaster et al., 1996) or indirectly inhibiting them via GABAergic interneurons (Jinde et al., 2012; Bui et al., 2018; Botterill et al., 2020). MCs in rodents send their axonal fibers across the dorsoventral axis of both the ipsilateral and contralateral DG (Buckmaster et al., 1996; Scharfman, 2016). These long-range projections of MCs actively regulate the bilateral DG activity along the longitudinal axis (Bauer et al., 2021; Fredes et al., 2021; Houser et al., 2021; Wang et al., 2021; Abdulmajeed et al., 2022). Interestingly, the manipulation of septal MCs has an impact on both spatial memory and anxiety (Bui et al., 2018; Wang et al., 2021). Furthermore, temporal MCs were discovered to exert an influence on contextual memory as well as anxiety (Fredes et al., 2021; Wang et al., 2021). These intriguing findings suggest that the distinctive features of MC connectivity may contribute to functional crosstalk between the dorsal and ventral portions, as well as between the bilateral DG (Amaral and Witter, 1989).

Despite the evolutionary similarity in intrinsic organization and primary function of the hippocampus, there are remarkable differences between species, especially in neuronal features and anatomical connectivity (Amaral et al., 1984; Rosene and Van Hoesen, 1987; van Dijk et al., 2016). Indeed, anatomical studies have shown that the DG has drastic differences across species in cell number, neurochemical content, and connectivity (Amaral et al., 1984, 2007; Seress et al., 2008; van Dijk et al., 2016). Recently, we and others have documented within-class heterogeneity of MCs depending on their longitudinal locations in the mouse hippocampus (M. Jeong et al., 2020; Botterill et al., 2021; Houser et al., 2021). Interestingly, septal and temporal mouse MCs differ in their axonal projections in the molecular layers of the mouse DG. However, it has not been explored whether anatomical features of mouse MCs are conserved across mammalian species.

With motivation from recent findings regarding the septotemporal heterogeneity of MCs and their distinct axonal projections in mice, we carried out a comparative analysis between mice and monkeys. In the present study, we used dual-fluorescence labeling of septal and temporal MCs in both species. We found that associational MC subpopulations are conserved across species. Here, we report conserved septotemporal heterogeneity within MCs across species, but a complete absence of commissural connectivity in the monkey DG, unlikely in mice. Despite the conserved septotemporal heterogeneity of MCs, we further found that septal MC axons innervate the inner molecular layer (IML) of the septal DG in monkeys, gradually expanding into the edge of the IML and sparsely even up to the middle (MML) and outer molecular layer (OML) through the temporal DG. This was unlike the dense projections in the MML of the temporal DG in mice, suggesting interspecies differences in the topographical organization of septal MC projections. This information provides a potential clue to addressing the anatomical and functional evolution of the hippocampal network in mammals.

## Materials and Methods

### Animals

#### Mouse

The Calcrl-Cre male TG mice (JAX stock, #023014) were imported from the Jackson (JAX) Laboratory. The transgenic mice were bred with C57BL/J mice (JAX) to obtain hemizygotes. Animals were group-housed, with 2–5 per cage, under a 12 h light/dark cycle (7 A.M.–7 P.M.) and provided a standard diet (LabDiet) and water *ad libitum*. We began all of the animal experiments at the age of 8–15 weeks. All experimental procedures were approved by the Animal Care and Use Committee of the Daegu Gyeongbuk Institute of Science & Technology (DGIST, IACUC #20011503-03).

#### Monkey

Rhesus monkeys (*Macaca mulatta*) were obtained from Suzhou Xishan Zhongke Laboratory Animal and housed in individual indoor cages at the National Primate Research Center (NPRC) of the KRIBB, as described previously (Yeo et al., 2019). Animals were fed twice daily with commercially available monkey feeds (Harlan, USA) supplemented with various fruits and water *ad libitum*. The controlled environmental conditions were as follows: temperature, 24 ± 2°C relative humidity, 50 ± 5%; and a 12 h light/dark cycle. The experiment involved three female subjects, one for immunohistochemistry and the other two for stereotaxic injections of fluorescent protein. All subjects were 6 years of age. All experimental procedures described involving animal care and the use of nonhuman primates were approved by the Korea Research Institute of Bioscience and Biotechnology (KRIBB) Institutional Animal Care and Use Committee (KRIBB-AEC-21102). Experimental procedures were performed in accordance with national guidelines and complying with the Guidelines for the Care and Use of Laboratory Animals. All animals were monitored a minimum of two times daily and were provided appropriate veterinary care by trained personnel. The health of animals was monitored by the attending veterinarian consistent with the recommendations of the Weatherall Report. Animal health monitoring was performed by microbiological tests including B virus, simian retrovirus (SRV), simian immunodeficiency virus (SIV), simian virus 40 (SV40), and simian T-cell lymphotropic virus (STLV) once a year, as described previously (H. S. Jeong et al., 2018).

### Adeno-associated virus (AAVs) and stereotaxic surgery

#### AAVs

The following AAV stocks to express a fluorescence protein were used in this study: AAV1.CAG.FLEX.EGFP (Penn Vector Core), AAV2.CAG.FLEX.tdTmato (UNC Vector Core), AAV2.CamKIIa-EGFP, and AAV2-CamKIIa-mCherry (Addgene).

#### Mouse

Calcrl-Cre TG mice were deeply anesthetized with an intraperitoneal injection of Avertin (250 mg/kg) and placed into a stereotaxic apparatus (Angle Two, Leica Biosystems). Original AAV stocks were diluted in 1 × 10^12^ GC/ml with AAV dilution solutions (5% sorbitol in 1× PBS), and 500 nl was injected into either the dorsal hilus (dHil; −2.1 mm AP, ±1.4 mm ML, −1.95 mm DV) or ventral hilus (vHil; −3.3 mm AP, ±2.7 mm ML, −3.6 mm DV). The flow rate (200 nl/min) was controlled using a Nanopump controller (World Precision Instruments). The needle was left in the target region for an additional 5 min after the injection. Calcrl-Cre TG mice were allowed at least 3 weeks for surgical recovery. Any mice with abnormal recovery after stereotaxic surgery were killed and thus excluded from the analysis. All injections were verified histologically at the end of the experiments.

#### Monkey

Rhesus monkey was anesthetized via intraperitoneal injection of a cocktail mixture of ketamine (5 mg/kg) and atropine (0.02 mg/kg). The head was immobilized within a custom-built CT and MRI-compatible stereotaxic frame under isoflurane-induced anesthesia (1.5% in 2 L/min oxygen). The fiducial MRI marker was attached to the skull surface as a precise reference point. Then, a brain T1-weighted MRI was performed as a baseline reference. Baseline images were used to determine the stereotaxic coordinates of a targeted brain region such as the septal and temporal DG. Two circular craniotomies centered on the skull surface toward the targeted coordinates were performed in the ipsilateral hemisphere, ∼3–5 mm in diameter. A total of 10 μl AAV was injected using the Hamilton syringe and micro infusion pump by convection-enhanced delivery (CED; Yazdan-Shahmorad et al., 2018) into the septal (6.6 × 10^12^ GC/ml) and temporal DG (4 × 10^12^ GC/ml). An initial infusion rate of 0.2 μl/min was applied and increased sequentially to 0.4, 0.6, and 0.8 μl/min at 5 min intervals. A gadolinium-based MRI contrast agent was infused with the viral vector (1:200) in order to verify the correct injection site. After the infusion, the syringe needle was kept in the targeted region for 10 min before retraction and slowly removed from the brain. Vital signs, including heart rate, pO_2_, and body temperature were monitored during anesthesia throughout all surgical procedures. The monkey was allowed a recovery period of 4 weeks post-surgery.

### Brain tissue preparation

#### Mouse

All animals were anesthetized by intraperitoneal injection of Avertin (250 mg/kg) and perfused transcardially with PBS, followed by 4% paraformaldehyde (PFA). The brains were extracted and incubated in 4% PFA overnight at 4°C, before being transferred to 15% sucrose until they sank. Subsequently, they were transferred to 30% sucrose and left overnight at 4°C. The brains were horizontally cut into 40 μm sections using a Cryostat (CM3050S, Leica Biosystems).

#### Monkey

Animals were transcardially perfused with heparinized PBS followed by 4% PFA under deep anesthesia with intramuscular injection of a cocktail of ketamine (5 mg/kg) and atropine (0.02 mg/kg). The brains were extracted and sliced into 8 mm coronal slices using a custom-built monkey brain slicer. Brain slices were incubated in 4% PFA for 24 h at 4°C, before being transferred to 15% sucrose until they sank and then transferred to 30% sucrose for 72 h at 4°C. After dehydration of brain tissue, brain slices were embedded in Optimal Cutting Temperature (OCT) compound, and the embedded brains were frozen at −80°C. The embedding blocks were cut on a cryostat into 40 μm thick sections.

### Immunochemistry

For immunostaining, each slice was incubated with blocking buffer (1× PBS, 0.2% BSA, 4% normal goat serum, 0.3% Triton X-100) at room temperature for 1 h. Following blocking, sections were incubated overnight at 4°C with primary antibodies diluted in the blocking buffer. The primary antibodies used were as follows: anti-GluR2/3 (rabbit polyclonal, 1:100, catalog #ab1506, Millipore), anti-calretinin (rabbit polyclonal, 1:500, catalog #7699/3h, SWANT) for distinguishing MC subpopulations in both the mouse and monkey DG, anti-EGFP (rabbit polyclonal, 1:500, catalog #ab6556, Abcam), and anti-mCherry (rat monoclonal, 1:500, catalog #M11217, Thermo Fisher Scientific) to enhance the fluorescence intensity of each MC subpopulation's axons in the monkey DG. After incubation for 24 h, sections were washed three times with washing buffer (1× PBS, 0.3% Triton X-100) for 10 min each and then were incubated with either tyramide signal amplification (catalog #B40955, Thermo Fisher Scientific) for anti-GluR2/3 or Alexa Fluor-conjugated secondary antibodies (1:400, Thermo Fisher Scientific) for the remaining primary antibodies at room temperature for 3 h. The sections were washed three times, counterstained with DAPI, and mounted using ProLong Gold antifading mounting medium (catalog #P36930, Thermo Fisher Scientific).

### Confocal imaging

The sections were scanned using a confocal microscope (LSM 780/800, Zeiss) with a 20× objective lens under consistent imaging conditions. Images were acquired with a frame size of 1,024 × 1,024 pixels. In cases where the region of interest was too large to fit within a single image, tile scans, and *z*-stack images (∼15 μm) were acquired using ZEN software (Zeiss).

### Quantification of confocal images

#### Estimation of the relative ratio of MC subpopulations

GluR2/3, a pan-MC marker, and CRT, a temporal MC marker, were used to distinguish the septal and temporal MC subpopulations. In mice, horizontal sectioning of the hippocampus with 40 μm thickness yielded ∼50–60 slices. Based on the horizontal anatomy of the mouse DG, the septal DG exhibits significant morphological variation. In contrast, the ventral part remains relatively uniform (Paxinos and Franklin's *The Mouse Brain in Stereotaxic Coordinates Fourth Edition*). To prevent duplication of similar anatomy of the DG, the hippocampal slice was sampled every three to six sections (90–160 μm) of the septal DG (#1∼#3) and every eight to 12 sections (300–400 μm) of the temporal part (#4∼#8), which help to collect a series of representative DG sections along the dorsoventral axis of the mouse hippocampus. In monkeys, coronal hippocampus sections with 40 μm thickness yielded ∼308 slices. The hippocampal slices were sampled at intervals of 800–1,600 μm along the anteroposterior axis of the monkey hippocampus, considering the anatomical features of the monkey DG (The Atlas of the Rhesus Monkey Brain, Elsevier, Academic Press). In mice, the hilar region was defined as the area inside the GC layer extending to the end of the CA3c pyramidal cell layer. In monkeys, the hilar regions were defined between the GC layer and the CA4 field using GluR2/3 and calretinin immunoreactivity (Buckmaster and Amaral, 2001). MCs were identified as GluR2/3 positive neurons and large nucleus (DAPI stained) in the dentate hilus and counted in the series of sections across the septotemporal axis of the hippocampus. A rough estimation of septal and temporal MCs was obtained by multiplying the number of MCs in each representative section by the corresponding number of sections and then summing them.

#### Quantification of MC axons

In the series of confocal images representing the septotemporal axis of the hippocampus, we assessed axonal projection patterns from septal and temporal MCs. In each section, relative fluorescence intensity across molecular layers was measured using a line scan mode of ImageJ (NIH) in 150-pixel width. The hippocampal subregions and molecular layers were defined by the well-characterized laminar distribution pattern visualized with a nuclei-staining dye (DAPI; Amaral et al., 2007). The subdivisions of the molecular layer (inner, middle, and outer) were determined by dividing the total width of the molecular layer into equal thirds.

### Three-dimensional imaging

#### Clearing of the intact brain

For imaging of the whole hippocampal region, the posterior two-thirds of a whole postmortem mouse brain was dissected and polymerized in a 1× PBS solution containing 1% acrylamide (acrylamide:bis-acrylamide = 29:1) and 0.1% Azo-initiator (VA-044, Wako) overnight, followed by polymerization for 3 h at 37°C (X-Clarity polymerization system, Logos Biosystems). Polymerized tissues were cleared in an X-Clarity tissue clearing system II (Logos Biosystems) for 8 h at a current of 2 A, temperature of 37°C, and pump speed of 80  rpm. Cleared tissues were stored in a refractive index matching solution (50% sucrose, 20% urea) for imaging with light-sheet fluorescence microscopy.

#### Three-dimensional (3D) fluorescence imaging

For light-sheet fluorescence microscopy, we used the Ultramicroscope II from LaVision BioTec equipped with Olympus MVPLAPO 0.63× lens with dipping cap, NKT Photonics SuperK EXTREME EXW-12 white light laser, Andor Neo sCMOS camera (Thorlabs), and a customized sample holder. Scans were made at 0.63× magnification with a light-sheet numerical aperture adjusted at 0.073. For EGFP and tdTomato fluorescence proteins, excitation filters of 470/40, 560/25, emission filters of 525/50, and 620/60 were used. The scan step size was set at 3 μm, and both channels were obtained in two separate scans. For the image postprocessing and 3D image rendering, serial TIF image files were converted to an Imaris file format and analyzed with Imaris software (Bitplane).

### Experimental design and statistical analysis

**Figure 1.**
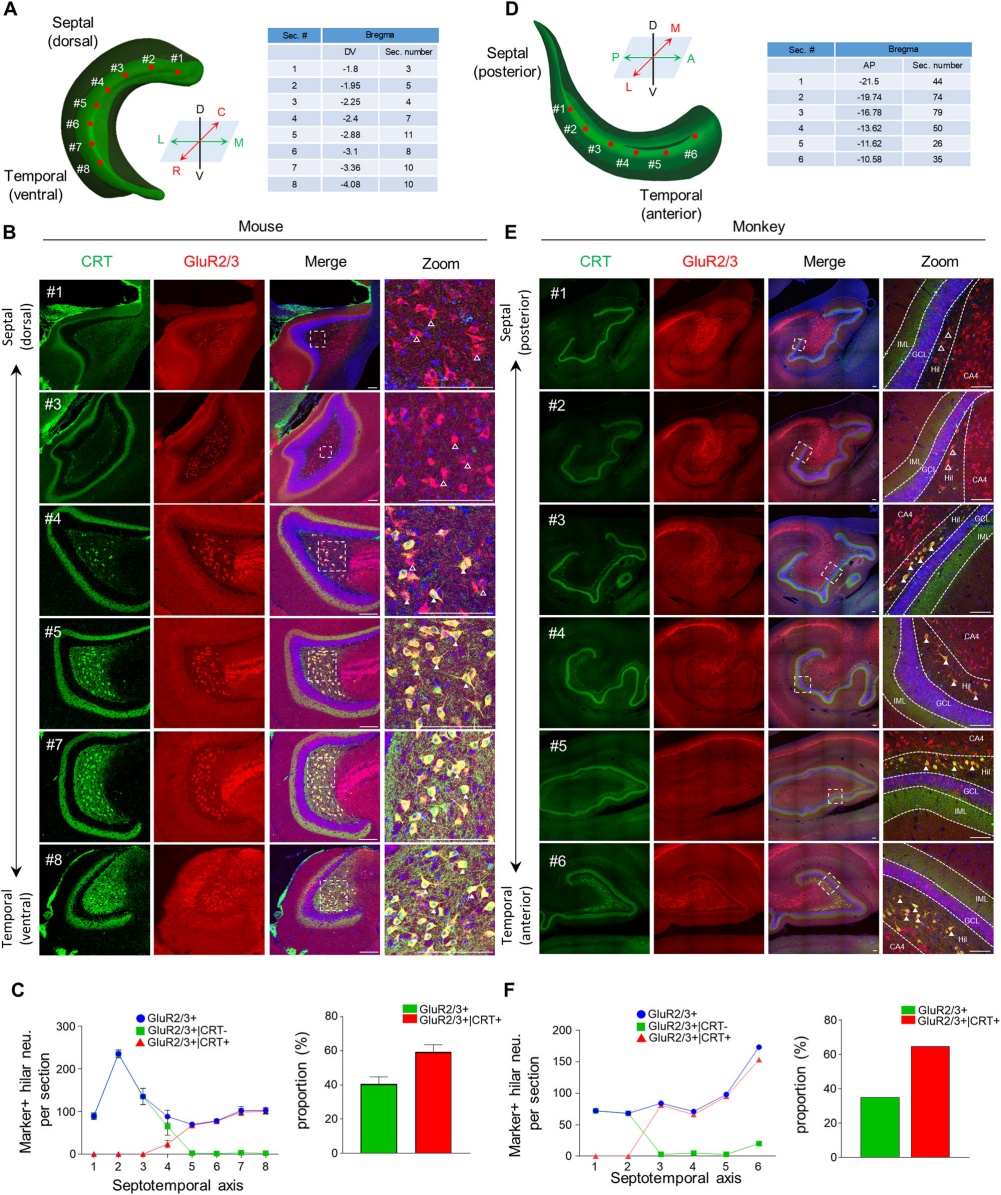
Two distinct MC subpopulations in mouse and monkey are spatially segregated along the septotemporal axis. ***A***, ***D***, Schematics depicting histological analysis across the septotemporal location in mouse (***A***) and monkey (***D***). ***B***, ***E***, Representative images showing GluR2/3 (red, pan-MCs marker) and CRT (green, temporal MCs marker) expressions in MCs along the septotemporal axis of the DG in mouse (***B***) and monkey (***E***). Open arrowheads represent only GluR2/3+ neurons, and filled arrowheads indicate colocalization of GluR2/3+ neurons with the CRT+ marker in the DG. ***C***, ***F***, Quantification of the spatial distribution of each MC subpopulation in mouse (***C***) and monkey (***F***). Septal and temporal MCs were determined based on marker pattern: GluR2/3+|CRT− as septal MCs and GluR2/3+|CRT+ as temporal MCs. The ratio for each MC subpopulation was determined by dividing the number of each subpopulation by the corresponding total number of MC subpopulations. IML, inner molecular layer; Hil, hilus; GCL, granule cell layer; D, dorsal; V, ventral; R, rostral; C, caudal; L, lateral; M, medial; A, anterior; P, posterior. Scale bars, 100 μm. Data are represented as mean ± SEM.

**Figure 2.**
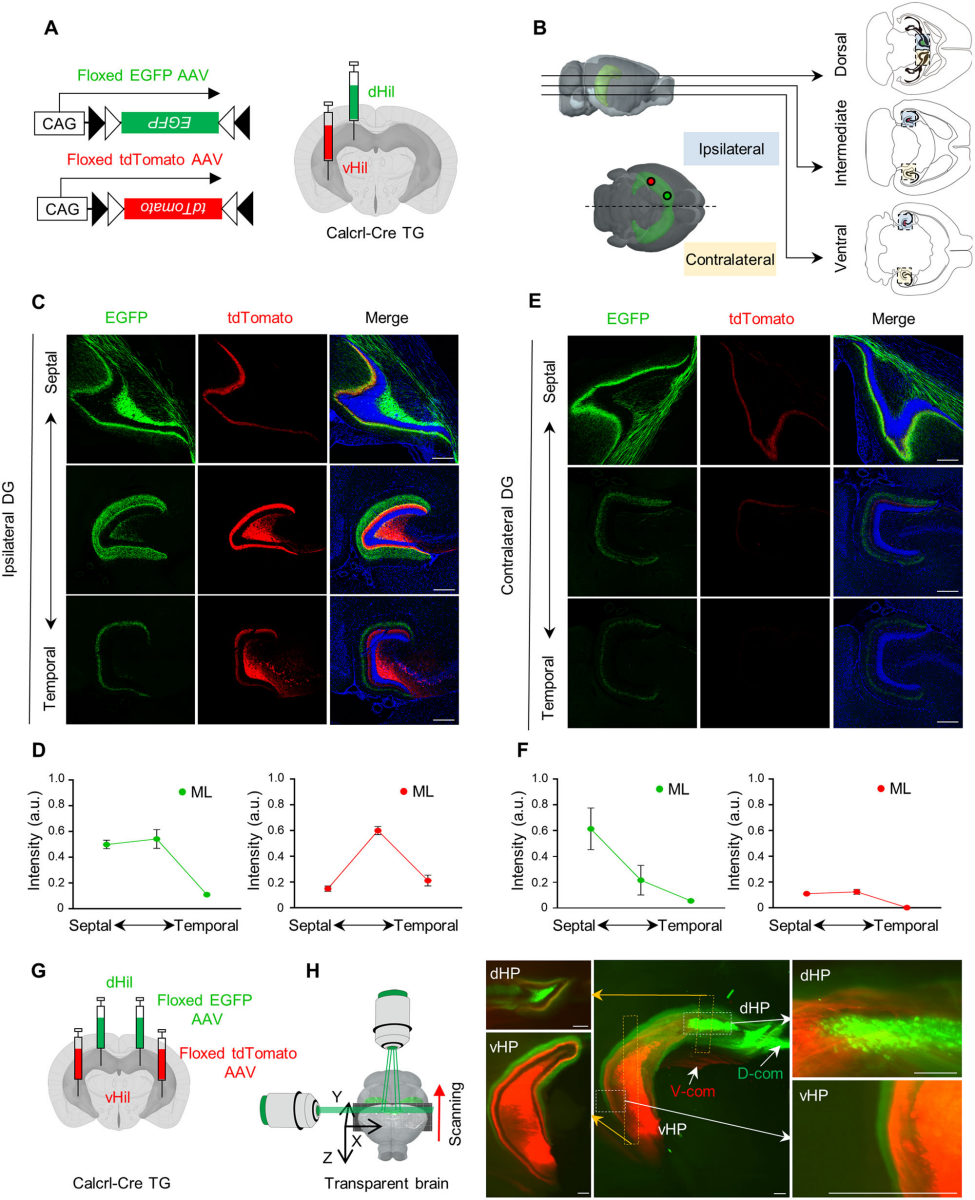
Septal and temporal MCs in mouse extend associational and commissural projections in the DG. ***A***, Schematic of viral injections in either the septal DG or temporal DG of Calcrl-Cre mice. Calcrl-Cre mice were unilaterally injected with Cre-dependent expressing EGFP and tdTomato virus into either the septal or the temporal DG, respectively. ***B***, Schematics depicting fluorescence imaging of the DG along the septotemporal axis of the DG in mouse. ***C***, ***E***, Representative images showing axonal projections of septal MCs (EGFP) and temporal MCs (tdTomato) in the ipsilateral (***C***) and contralateral (***E***) DG. ***D***, ***F***, Quantitative analysis of ipsilateral (***D***) and contralateral (***F***) of MC axons in the molecular layers. The EGFP+ septal MC axons or tdTomato+ temporal MC axons were measured across the molecular layers (mouse, *n* = 3). Axonal projections of septal MCs (EGFP) in the ipsilateral DG, *F*_(2,6)_ = 26.49, *p* = 0.0011; axonal projections of temporal MCs (tdTomato) in the ipsilateral DG, *F*_(2,6)_ = 55.8, *p* = 0.0001; axonal projections of septal MCs (EGFP) in the contralateral DG, *F*_(2,6)_ = 6.274, *p* = 0.0339; axonal projections of temporal MCs in the contralateral DG, *F*_(2,6)_ = 33.71, *p* = 0.0005. ***G***, Schematic of viral injections in either the septal DG or temporal DG of Calcrl-Cre mice. Calcrl-Cre mice were bilaterally injected with Cre-dependent expressing EGFP and tdTomato virus into either the septal or the temporal DG, respectively. ***H***, Three-dimensional (3D) fluorescence imaging was conducted on a virus-injected transparent brain using light-sheet fluorescence microscopy. This imaging technique allowed for a detailed three-dimensional rendering of MC projections in the hippocampus. dHil, dorsal hilus; vHil, ventral hilus; ML, molecular layer; dHP, dorsal hippocampus; vHP, ventral hippocampus; D-com, dorsal commissural fibers; V-com, ventral commissural fibers; D, dorsal; V, ventral; R, rostral; C, caudal; L, lateral; M, medial. Scale bar, 300 μm. Data are represented as mean ± SEM.

**Figure 3.**
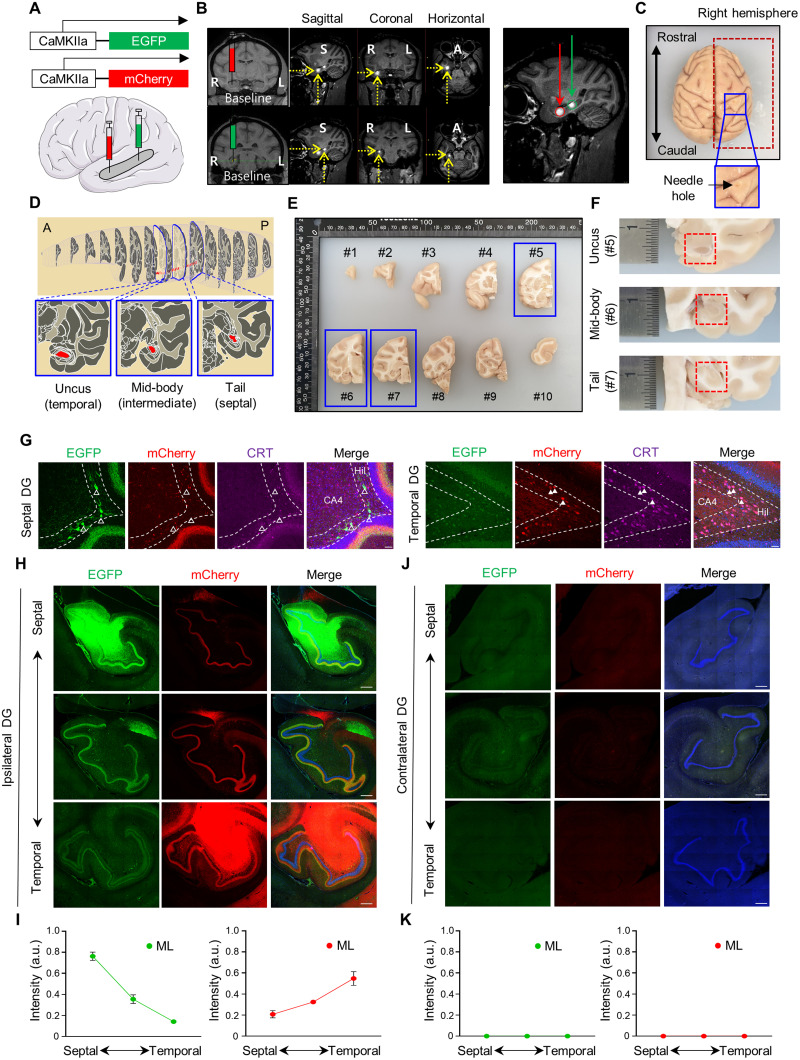
Septal and temporal MCs in monkey make associational projections in the ipsilateral DG, but not commissural projections in the contralateral DG. ***A***, Schematics for viral injections in a monkey. The septal and the temporal DG of a rhesus monkey were injected with AAVs expressing EGFP and mCherry under CaMKIIa promotor, respectively. ***B***, MRI imaging with multiple planes showing viral injection in a location-specific manner. To visualize the injection site, an MRI contrast agent was infused with AAVs. ***C***, Imaging showing unilateral viral-injected monkey brain. ***D***, Schematics for the septotemporal axis of the monkey DG. ***E***, Viral-injected monkey brain were cut with a coronal plane interval of 8 mm. The number is an order of the anteroposterior axis by coronal plane. Based on the spatial distribution of MCs, we chose the brain block of #5, #6, and #7. ***F***, Image showing the position of the DG in the uncus (temporal), mid-body (intermediate), and tail (septal) of the hippocampal formation in the monkey. ***G***, Representative image showing fluorescent protein expressions in septal and temporal MCs of the monkey DG. CRT, a temporal MC marker (purple), labeled neurons were colocalized with mCherry-expressing neurons in the temporal DG, but not with EGFP-expressing neurons in the septal DG. Scale bar, 50 μm. Open arrowheads indicate EGFP+ neurons without the CRT+ marker (septal MCs), while filled arrowheads indicate colocalization of mCherry+ neurons with the CRT+ marker in the DG. ***H***, ***J***, Representative images showing axonal projections of septal and temporal MCs in the ipsilateral (***H***) and contralateral (***J***) DG. EGFP-labeled axons from septal MCs and mCherry-labeled axons from temporal MCs were observed along the septotemporal axis of the ipsilateral DG (***H***), but not of contralateral DG (***J***). Labeling of septal MC, bregma: −19.35 mm, ∼80% in the hilus; labeling of temporal MC, bregma: −11.70 mm, ∼80% in the hilus. ***I***, ***K***, Quantitative analysis of axonal projections density of septal and temporal MCs at the molecular layers of the ipsilateral (***I***) and the contralateral (***K***) DG along the septotemporal axis. Relative fluorescence intensity was measured randomly at three different locations of the molecular layers (monkey; *n* = 1; replicate, 3). Axonal projections of septal MCs (EGFP) in the ipsilateral DG, *F*_(2,6)_ = 89.02, *p* < 0.0001; axonal projections of temporal MCs (mCherry) in the ipsilateral DG, *F*_(2,6)_ = 16.58, *p* = 0.0036; axonal projections of septal MCs (EGFP) in the contralateral DG, *F*_(2,6)_ = 0.4705, *p* = 0.6459; axonal projections of temporal MCs in the contralateral DG, *F*_(2,6)_ = 1.814, *p* = 0.2420. Scale bar, 300 μm. ML, molecular layers. Data are represented as mean ± SEM.

**Figure 4.**
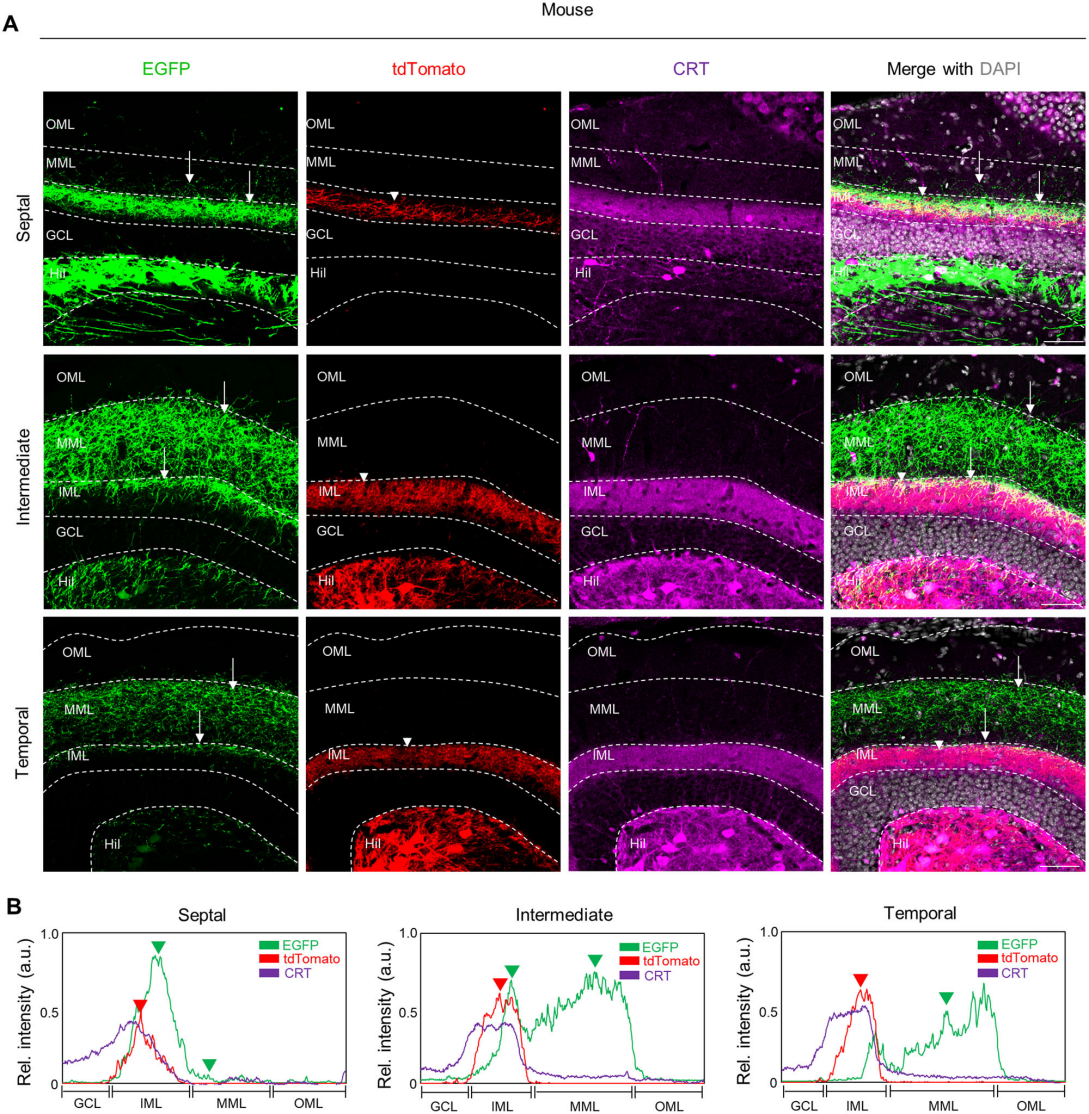
Septotemporal heterogeneity of MCs in their axonal projections in the DG molecular layers in the mouse. ***A***, Representative images showing axonal projections of septal and temporal MCs along the septotemporal axis of the DG in mouse. Scale bar, 50 μm. ***B***, The distribution pattern of axonal fibers from septal (EGFP) and temporal (tdTomato) MCs in each molecular layer of the mouse. CRT a temporal MC marker (purple) was colocalized with tdTomato-labeled temporal MC soma (red) and their axonal fibers (tdTomato), but not with septal MCs (EGFP). The fluorescence intensity of the axonal projections of the MC subpopulations and CRT expression was measured using line scanning. Arrows and arrowheads indicate axonal fibers from septal and temporal MCs in the DG, respectively. GCL, granule cell layer; IML, inner molecular layer; MML, middle molecular layer; OML, outer molecular layer.

**Figure 5.**
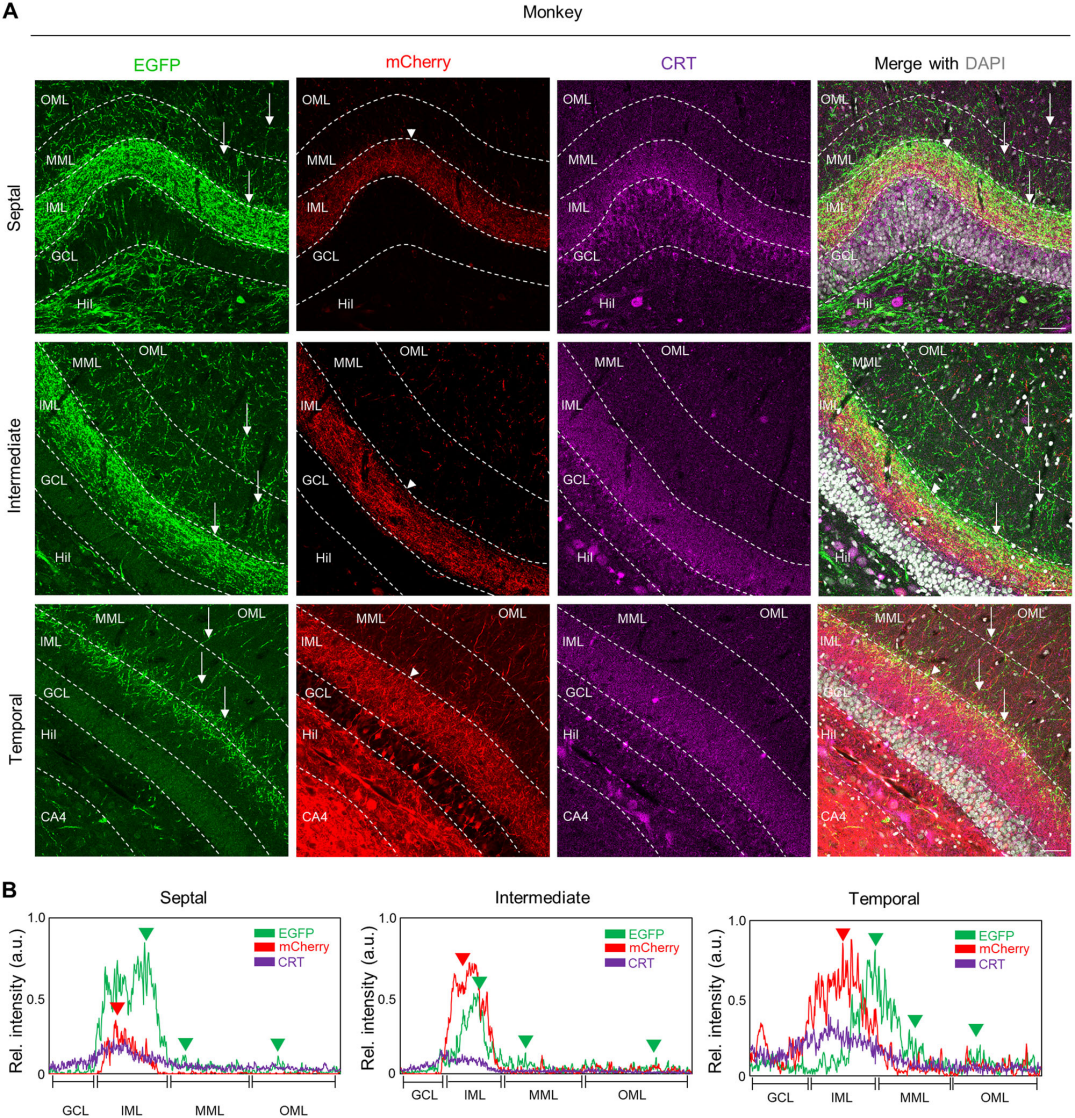
Septotemporal heterogeneity of MCs in their axonal projections in the DG molecular layers in the monkey. ***A***, Representative images showing axonal projections of septal and temporal MCs along the septotemporal axis of the DG in monkey. Scale bar, 50 μm. ***B***, The distribution pattern of axonal fibers from septal (EGFP) and temporal (mCherry) MCs in each molecular layer of the monkey DG. CRT a temporal MC marker (purple) was colocalized with tdTomato-labeled temporal MC soma (red) and their axonal fibers (mCherry), but not with septal MCs (EGFP). The fluorescence intensity of the axonal projections of the MC subpopulations and CRT expression was measured using line scanning. Arrowheads indicate a high density of axonal fibers from septal (EGFP) and temporal (tdTomato) MCs at each molecular layer. GCL, granule cell layer; IML, inner molecular layer; MML, middle molecular layer; OML, outer molecular layer. For axonal projection mapping in another monkey subject, please see Extended Data Figure 5-1.

This study aimed to compare anatomical features in the spatial distribution and axonal projections of MCs along the septotemporal axis between mouse and monkey. To investigate this, we designed the following three experiments: Experiment 1: Comparative analysis of spatial distribution of MC subpopulations between mouse and monkey. In this experiment, we performed immunohistochemistry to classify MC subpopulations using GluR2/3, a pan-MC marker, and calretinin, a temporal MC marker, in both mice (*n* = 5) and monkey (*n* = 1; Fig. 1). Experiment 2: Characterization of rodent MC subpopulations in axonal projections (*n* = 3). In this experiment, we employed a Cre-dependent dual AAV approach based on the spatial distribution of MC subpopulations to examine axonal projections from MC subpopulations to the septotemporal axis of the DG (Fig. 2). Experiment 3: Characterization of monkey MC subpopulations in axonal projections (*n* = 1). In this experiment, we used rhesus monkey to label MC subpopulations across the septotemporal axis of the DG using the dual AAV approach (CaMKII-dependent expression of fluorescent proteins; Fig. 3). With these three experiments, we observed the conservation of septotemporal heterogeneity of MCs across species but noted interspecies differences in the axonal projection patterns of septal MCs along the long axis of the DG (Figs. 4, 5). All data are represented as mean ± standard error of the mean (SEM). Statistical parameters and analysis performed can be found in the figure legends. Statistical analyses were performed using Prism 7.0a (GraphPad). Axonal projections of septal and temporal MCs were examined by one-way ANOVA. Post hoc analyses were conducted using Tukey's test. Statistical significance was set at *p* < 0.05.

10.1523/ENEURO.0151-24.2024.f5-1 Figure 5-1 Septotemporal heterogeneity of MCs in their axonal projections in the molecular layers of the DG in another monkey subject. **A**, Schematics for viral injections in a monkey. The septal and temporal DG of a rhesus monkey were injected with AAVs expressing EGFP and mCherry under the CaMKIIa promoter, respectively. MRI imaging with multiple planes shows viral injection in a location-specific manner. An MRI contrast agent was co-infused with AAVs to visualize the injection site. **B**, Representative images showing axonal projections of septal and temporal MCs along the septotemporal axis of the DG in the monkey. Labeling of septal MC, Bregma: -20.50 mm, approximately 10% in the hilus; labeling of temporal MC, Bregma: -12.60 mm, approximately 80% in the hilus. The scale bars represent 500 μm for large-scale images and 50 μm for zoomed images. Distribution pattern of axonal fibers from septal (EGFP) and temporal (mCherry) MCs in each molecular layer of the monkey DG. Arrows indicate axonal fibers from septal (EGFP) at each molecular layer. Hil, hilus; GCL, granule cell layer; IML, inner molecular layer; MML, middle molecular layer; OML, outer molecular layer. Download Figure 5-1, TIF file.

**Movie 1.** Axonal projections of septal and temporal MCs along the septotemporal axis of the mouse hippocampus. Calcrl-Cre mouse was bilaterally injected with Cre-dependent EGFP and tdTomato-expressing AAVs into the dorsal and ventral DG. The movie illustrates the axonal projections of EGFP-labeled (septal MCs) and tdTomato-labeled (temporal MCs) neurons in the mouse hippocampus. The movie showed axonal projections of MC subpopulations in the intact mouse hippocampus from various angles, including horizontal, vertical, and detailed views. The scale bar was embedded in the left corner of the image. [View online]

## Results

### Spatial segregation of two distinct MC subpopulations along the septotemporal axis of the DG in mouse and monkey

The hippocampus is organized into multiple functional domains, often exhibiting sharply demarcated borders or linear gradient patterns along the septotemporal axis (Strange et al., 2014). Here, we examined spatial distribution patterns of each MC subpopulation along the septotemporal axis by using two species-conserved MC markers: glutamate receptor subunits 2/3 (GluR2/3) as a pan-MC marker (Leranth et al., 1996; Fujise and Kosaka, 1999) and calretinin (CRT), which serves as the temporal MC marker (Blasco-Ibáñez and Freund, 1997; Seress et al., 2008). Based on these two species-conserved MC markers, we could classify MCs into two distinct subpopulations: septal MCs as GluR2/3+|CRT− populations and temporal MCs as GluR2/3|CRT+ ones. In mice, brains were sectioned in the horizontal plane along the septotemporal axis of the mouse DG to examine the spatial distribution of MC subpopulations (Fig. 1*A*). We observed that the septal MCs were exclusively located in one-third of the septal DG (bregma: −1.7 to −2.4 mm, dorsoventral coordinate; Fig. 1*B*,*C*). Septal (GluR2/3+|CRT−) and temporal (GluR2/3+|CRT+) MCs were spatially segregated with a narrow borderline (bregma: −2.4 mm, dorsal-ventral coordinate) where the two populations intermingled (Fig. 1*B*,*C*). Temporal MCs were distributed in two-thirds of the temporal DG (bregma: −2.4 to −4.08 mm, dorsoventral coordinate; Fig. 1*B*,*C*). Next, to examine the distribution of MC subpopulations in monkey, brains were sectioned in the coronal plane (Fig. 1*D*). Differently from the mouse DG, the hilus region in the monkey exists as the narrow zone between the CA4 region and the GC layer (Fig. 1*E*). Septal MCs (GluR2/3|CRT−) were predominantly distributed in the septal DG (bregma: −21.5 to −19.74 mm, anteroposterior coordinate; Fig. 1*E*,*F*). Temporal MCs were widely distributed in the temporal DG (bregma: −19.74 to −10.58 mm, anteroposterior coordinate), suggesting a similar distribution of MC subpopulation along the septotemporal axis between species (Fig. 1*E*,*F*). We investigated the relative distribution of MC subpopulations within the DG of both mice and monkey, focusing on the septotemporal axis. Our approximate findings indicate that in mice, the MCs are divided into 40% septal and 60% temporal subpopulations (Fig. 1*C*, right). Similarly, in monkey, the MCs present a distribution with a composition of 35% septal and 65% temporal subpopulations (Fig. 1*F*, right). Temporal MCs outnumber the septal ones by 1.5–1.8 times in both species (Fig. 1*C*,*F*), suggesting that the relative proportion is conserved between mouse and monkey. Taken together, MCs display species-conserved septotemporal heterogeneity in terms of a classical MC marker, CRT expression.

### Associational and commissural projections of septal and temporal MCs in the mouse DG

Based on the histological distribution of the classical MC marker CRT, MCs are known to project to the IML along the septotemporal axis of the DG (Blasco-Ibáñez and Freund, 1997). However, CRT expression in MCs specifically is limited to the temporal DG and thus does not represent the structural features of MCs in the septal DG. Based on the spatial distribution of each MC subpopulation pattern (Fig. 1*B*), we compared the axonal projection patterns of septal MCs and temporal MCs in an animal model by unilateral injection of either AAV2-CAG-DIO-EGFP or AAV2-CAG-DIO-tdTomato into the septal and temporal DG of Calcrl-Cre mice, respectively (Fig. 2*A*). Fluorescence protein expression in AAV-injected Calcrl-Cre mice was highly specific in MCs (septal MCs, 95 ± 1.4%; temporal MCs, 95 ± 1.7%) but was undetectable in GCs and other interneurons such as parvalbumin-positive basket cells and hilar perforant path-associated cells, consistent with the literature (Botterill et al., 2020, 2021; Oh et al., 2020; data not shown). We first examined the associational/commissural projections of each MC subpopulation across the septotemporal axis of the DG. We visualized horizontal sections across the septotemporal axis of the DG (Fig. 2*B*). In the ipsilateral DG, tdTomato-labeled axonal fibers of temporal MCs were found exclusively in the IML of the DG, which is consistent with the histological pattern of CRT expression (Blasco-Ibáñez and Freund, 1997; Fig. 2*C*). In contrast, EGFP-labeled axons of septal MCs were found mainly in the IML of the septal DG (Fig. 2*C*). Strikingly, the EGFP-labeled axons originating from septal MCs exhibited widespread distribution across molecular layers, with a noticeable prevalence toward the temporal DG (Fig. 2*C*,*D*), indicating distinct axonal projections between septal and temporal MCs. Commissural axons of either septal or temporal MCs were found in the contralateral DG, and these layer-specific projections were highly consistent in the contralateral DG, despite the reduced density of axonal fibers as compared with that in the ipsilateral DG (Fig. 2*E*,*F*).

For a holistic visualization of axonal projections of MCs in an intact hippocampus, we took advantage of the CLARITY technique (Chung et al., 2013). We bilaterally injected either Cre-dependent AAV2-CAG-DIO-EGFP or AAV2-CAG-DIO-tdTomato into the septal and temporal DG, respectively (Fig. 2*G*). Our three-dimensional rendering images showed proper segregated labeling of septal and temporal MCs along the septotemporal axis of the DG (Fig. 2*H*). Septal MCs and temporal MCs project to the contralateral DG through the dorsal and ventral hippocampal commissure, respectively (Fig. 2*H*, Movie 1). Aligning with confocal images of hippocampal sections, a holistic view of MC projections displayed clear layer specificity along the septotemporal axis of the DG (Fig. 2*H*, Movie 1). Furthermore, we observed that longitudinal axons from septal MCs and temporal MCs collectively cover ∼80% of the entire DG (Fig. 2*H*), underscoring the structural significance of MCs for the functional association of the entire DG across the longitudinal axis. Taken together, septal and temporal MCs in mice exhibit discrete heterogeneity in their associational and commissural projections along the longitudinal axis of the bilateral DG.

### Associational projections of septal and temporal MCs, but absence of commissural projections, in the monkey DG

To investigate whether distinct axonal projections of MC subpopulations in mice are conserved in monkey, we used a similar viral approach as the mouse model. In contrast to the mouse, the monkey model is challenged to target cell-type specificity in the DG; thus we sought to minimize the cell-type specificity issue. To this end, we used the CaMKII promoter-driven expressing fluorescence proteins for selectively targeting excitatory neurons. A monkey was injected with a contrast agent mixed AAVs (either AAV2-CaMKIIa-EGFP or AAV2-CaMKIIa-mCherry) into the septal and temporal DG, respectively (Fig. 3*A*). The MRI imaging of the monkey's brain along the multiple views showed segregated septotemporal expressions of the contrast agent in the DG (Fig. 3*B*). Four weeks post stereotaxic surgery, the monkey was killed (Fig. 3*C*), and we examined long-range projections of MC subpopulations across the anteroposterior axis of the monkey. First, the brain was cut in the coronal plane with an interval of 8 mm, and the ipsilateral and the contralateral hippocampal sections were collected along the septotemporal axis based on the distribution of MC subpopulations (Figs. 1*E*, 3*D–F*). To verify whether each MC subpopulations were targeted along the septotemporal axis of the DG, the sections were processed for CRT immunohistochemistry. We observed that the mCherry+ neurons in the temporal DG colocalized with CRT a temporal MC marker, whereas EGFP+ neurons in the septal DG did not (Fig. 3*G*). In the ipsilateral DG, EGFP+ (septal MC) and mCherry+ (temporal MC) axons substantially extended associational projections along the septotemporal axis of the DG as in mice (Fig. 3*H*). The relative density of axonal fibers from septal and temporal MCs gradually decreased with distance from their cell body origins in the DG (Fig. 3*I*). In addition, we could not detect EGFP+ axonal fibers from septal MCs in the temporal pole of the DG (data not shown), implying that the associational fibers may reach <70% of the entire DG, which is largely consistent with previous report (Botterill et al., 2021; Houser et al., 2021). In sharp contrast to the mouse, the commissural projections from either septal or temporal MCs are barely visible in the contralateral DG of the monkey (Fig. 3*J*,*K*). Even with the high-resolution imaging, we were unable to detect mCherry+ and EGFP+ axonal fibers in the molecular layers of the contralateral DG (data not shown), indicating an absence of bilateral DG connectivity via MCs. Our data suggest that associational projections of MCs are conserved across species, with the notable exception of their commissural projections in monkey.

### Species difference in topological projections pattern of septal MCs in the molecular layers of the DG

Next, we analyzed axonal projections of MC subpopulations to the molecular layers in both mouse and monkey. AAV-injected hippocampal sections were immunolabeled with anti-CRT to visualize axons of temporal MCs. In mouse, tdTomato+ axons of temporal MCs are presented in the IML across the septotemporal axis of the DG, which were colocalized with CRT. In contrast, EGFP+ axons of septal MCs were found mainly in the IML and to a lesser extent in the MML of the septal DG (Fig. 4*A*,*B*). The relative density of septal MC axons in these molecular layers gradually diffused from IML to the MML along the septotemporal axis, which is a distinct pattern as compared with temporal MC axons.

In monkey, the mCherry+ axons of temporal MCs terminate the IML across the septotemporal axis of the DG, resembling axonal projection patterns observed in CRT expression and temporal mouse MCs (Fig. 5*A*,*B*). EGFP+ axons of septal MCs mainly project in the IML of the septal DG. Notably, as axons of septal MCs extended toward the temporal DG, their axons innervate the edge of IML and sparsely into the MML and OML (Fig. 5*A*,*B*), suggesting distinct axonal projections between septal and temporal MCs. Remarkably, mouse septal MCs project their axons expanding to the IML–MML toward temporal DG, while monkey septal MCs innervate their axons more in the IML and sparsely in MML–OML in the temporal DG. The axonal projection patterns of each MC subpopulation in the DG are consistent with those observed in another monkey subject (Extended Data Fig. 5-1). While the intrinsic heterogeneity of septal and temporal MCs is conserved across species, these distinct axonal projections are significantly different between species. Collectively, these results suggest intrinsic heterogeneity within MCs in axonal projections in monkey, despite interspecies differences in the topological organization of MCs.

## Discussion

MCs play a crucial role in the DG circuitry through associational and commissural connectivity in rodents. While an extensive body of work has revealed intrinsic heterogeneity of MCs along the septotemporal axis in rodents, our understanding of this heterogeneity in primates is yet to be explored. In this study, we investigated the neuroanatomical feature of MCs in the monkey DG in comparison with mouse DG. We found that two distinct subpopulations of MCs are spatially segregated along the septotemporal axis of the DG, which are conserved across the two species. Our dual fluorescence labeling approach revealed that both septal and temporal MCs in monkey project their associational fibers along the longitudinal axis of the ipsilateral DG, as in mouse. In contrast, these two subpopulations in monkey do not send commissural fibers to the contralateral DG, differently from those in mouse. Furthermore, we observed that septal and temporal MCs make differential axonal projections to the DG molecular layers in both species. Despite the conserved heterogeneity of MCs across species, there are notable differences in the topological organization of septal MC projections in the molecular layers between two species. Taken together, our data provide a potential clue to addressing functional differentiation along the septotemporal axis of the DG, as well as the anatomical evolution of the DG circuit in mammals.

### Species-conserved heterogeneity of septal and temporal MCs

The hippocampus undergoes functional differentiation along the septotemporal axis, a phenomenon attributed to variances in afferent/efferent connectivity and gene expression in principal cell types (CA1, CA3, and DG) (Strange et al., 2014; Cembrowski and Spruston, 2019). Previous studies have suggested that species-conserved MC marker calretinin in temporal MCs exhibit their axons in the IML of the DG (Fujise et al., 1997; Seress et al., 2008). Consequently, it has been generally assumed that MCs project to the IML throughout the longitudinal axis of the DG in mice and monkey. Recently, we and others have demonstrated the distinct pattern of axonal projections between septal and temporal MCs in mice (M. Jeong et al., 2020; Botterill et al., 2021; Houser et al., 2021). In this study, we observed a conservation of anatomical heterogeneity within MCs across species. We found that MC subpopulations spatially are segregated along the septotemporal axis of the DG in both mouse and monkey (Fig. 1). Based on their distribution of MC subpopulations, we showed that temporal MC axons terminate the IML throughout the septotemporal axis of the mouse and monkey DG (Figs. 2, 3) consistent with previous reports (Seress et al., 2008). Notably, septal MCs in monkey project to the IML of the septal DG and expand their axons into the edge of the IML and sparsely into the MML and OML in the temporal DG, suggesting distinct axonal projections of MC subpopulations along the septotemporal axis of the monkey DG (Fig. 5). Interestingly, we could observe similar patterns of MC projections along the septotemporal axis of the DG in the previous report that has examined the DG connectivity in cynomolgus monkey using anterograde tracing dye (Kondo et al., 2008). In line with this observed heterogeneity, mounting evidence has suggested that septal MCs are differentiated from temporal MCs in the DG across anatomical, transcriptional, and physiological properties in rodents. Specifically, (1) septal MCs exhibit complex thorny excrescences compared with temporal MCs (Jinno et al., 2003; Houser et al., 2021), (2) septal MCs and temporal MCs show transcriptional heterogeneity (Cembrowski et al., 2016; M. Jeong et al., 2020), (3) septal MCs have higher excitatory postsynaptic potential frequency and amplitude than temporal MCs (Jinno et al., 2003), and (4) septal and temporal MCs are significantly different effect on cognitive and affective behaviors in rodents (Fredes et al., 2021; Wang et al., 2021). While we found the existence of two MC subpopulations with distinct axonal projections in monkey, we are yet to determine whether these two populations display considerable differences in their morphological, transcriptional, and electrophysiological properties. Nonetheless, it is presumable that septal and temporal MCs in monkey may exhibit substantial differences in their neuronal features as characterized in mouse and thereby contribute differentially to cognitive and affective behaviors.

### Species difference in MC projections to the molecular layers of the DG

While the hippocampus exhibits evolutionary similarities across species, there are remarkable differences in morphological features, particularly in primates (Seress, 2007). In monkey, MCs penetrate their dendrite into molecular layers, a feature not observed in rodents (Frotscher et al., 1991), suggesting that monkey MCs receive substantial perforant path input. Our study demonstrates remarkable differences in axonal projections of MCs along the septotemporal axis of the DG. Temporal MCs in monkey project to the IML of the DG along the septotemporal axis, consistent with mouse study. Intriguingly, septal MCs innervate their axons mainly in the IML and sparsely in the MML–OML in the temporal DG, in contrast to the dense projections of the MML of the temporal DG from mouse septal MCs. Additionally, while septal and temporal MCs in mice exhibit extensive associational and commissural projections, our findings revealed that septal and temporal MCs in monkey have associational projections but lack commissural ones. Collectively, our study suggests significant differences in neuronal connectivity specifically in topological axonal patterns and commissural projections of MCs between species.

The DG exhibits a laminar organization of input, comprising IML, MML, and OML (Amaral et al., 2007), and these specific laminar inputs may be associated with the differential regulation of DG activity. Specifically, while the lateral entorhinal cortex (projecting to OML in the DG) triggers monosynaptic inhibition of mature GCs by adult-born GCs, the medial entorhinal cortex (projecting to MML in the DG) elicits excitatory responses in mature GCs by them (Luna et al., 2019). MCs in the DG are crucial for regulating the activity of GCs and play a significant role in mediating DG-dependent functions (Scharfman, 2016; Bui et al., 2018; Botterill et al., 2020; Fredes et al., 2021; Wang et al., 2021). In mice, temporal MCs generally terminate in the IML, and these activations induce a homogeneous effect on DG activity across the septotemporal extent of the DG (Bauer et al., 2021; Fredes et al., 2021). Differently from temporal MCs, septal MC in mouse project to the IML of the septal DG and expand their axons in the MML of the temporal DG, suggesting a differential effect on the septal and the temporal DG (Houser et al., 2021; Abdulmajeed et al., 2022). These observations have suggested that switching the axonal projections within molecular layers of the DG could potentially result in differential targets on GCs and GABAergic interneurons. Our data demonstrate interspecies differences in the topological organization of septal MCs to the molecular layers of the DG, implying a potential differential regulation of GC activity along the septotemporal axis. Therefore, it is important to consider the differences between species when studying the structure and function of neural circuits in the primate hippocampus.

The comparative anatomical analysis between mouse and monkey brains is important for understanding the evolutionary mechanisms of circuits and their functions. In this study, our anterograde tracing revealed the absence of commissural projections from both MC subpopulations in monkey to the contralateral DG. This observation aligns with findings from earlier tracing studies in monkeys, wherein only a small number of neurons were labeled in the contralateral uncal region of the monkey DG, supporting the notion of considerably restricted commissural projections of MCs in monkeys (Amaral et al., 1984; Demeter et al., 1985). Intriguing studies have shown that mice with transected ventral hippocampal commissural fibers exhibit asymmetry in postsynaptic receptor subunit expressions and physiological functions (LTP) in CA1, suggesting hippocampal lateralization (Kawakami et al., 2003). Thereby, it is plausible that the absence of commissural projections may support strong hippocampal lateralization in primates. Brain lateralization manifests as asymmetry at molecular and physiological levels, as well as differences in task-related activities between hemispheres, particularly in humans and nonhuman species (Rogers et al., 2004; Jordan, 2020; Nemati et al., 2023). This phenomenon may help avoid the duplication of brain functions and enhance cognitive capacity (Rogers et al., 2004; Rogers, 2021). Future studies are warranted on the functional roles related to the evolutionary regression of commissural projections of MCs in primates.

The lamina specificity in the hippocampus is governed by a complex interplay of axonal guidance molecules in development gradients and competition (Förster et al., 2006). Our study reveals that septal MCs in both mice and monkeys innervate the MMLs of the DG, extending toward the temporal DG. However, temporal MCs are notably restricted to the IML of the DG (Figs. 4, 5). Interestingly, organotypic slice culture studies have demonstrated that lesioning the entorhinal cortex leads to a wide expansion of temporal MCs axons, extending up to the MML and OML of the DG, suggesting that entorhinal cortex neurons exert a regulatory influence on temporal MCs, possibly through axonal competition (Del Turco et al., 2019). The ephrins/Eph receptors family plays a critical role in axonal guidance during hippocampal development. Specifically, EphA5-expressing neurons from the entorhinal cortex primarily target the MML and OML of the DG, while their ligand, ephrin-A3, is predominantly localized in the IML of the DG. This interaction between EphA5 receptors on entorhinal axons and ephrin-A3 in the IML facilitates a repulsive response, guiding the axons away from the IML of the DG (Stein et al., 1999). Our previous study has demonstrated differential expression of axonal guidance molecules in septal and temporal MCs in mice, implying their involvement in the specific axonal pathfinding of MC subpopulations (M. Jeong et al., 2020). It is plausible that the laminar specificity of septal and temporal MCs axons arises from a combination of factors including the axonal competition and septotemporal gradient of guidance molecules with the entorhinal cortex. On the other hand, our data showed a lack of commissural projections of MC subpopulations in primates. Commissural connections between hemispheres are organized and regulated by various molecules, including specific axon guidance molecules (Lindwall et al., 2007). During hippocampal development in mice, *netrin-1* exhibits transient localization in the target regions of commissural pathways, including CA1, CA3, and the DG. The netrin-1 receptor, DCC (Deleted in Colorectal Cancer), is expressed in the dentate hilus and CA3 regions, which serve as origins for projections to the contralateral hippocampus (Steup et al., 2000). Notably, Netrin-1-deficient mice show defects in the formation of the hippocampal commissure (Serafini et al., 1996). Further exploration of the molecular mechanism underlying interspecies of MC development is warranted.

### Limitations of study

Our present study has several limitations that warrant acknowledgment. Firstly, variations in the patterns of septal MC projections among animals could exist, depending on the location and extent of the AAV injections, which should be considered when interpreting the results. Secondly, our viral strategy in the monkey DG might inadvertently label GCs, CA4, and CA3 pyramidal cells alongside MCs, potentially influencing the clear identification of MC axonal projection patterns. Overall, while our study provides valuable insights into conserved and divergent features of MCs in the DG of mice and monkeys, these limitations should be taken into account when interpreting and generalizing the results from primates. Further research with larger sample sizes and more selective labeling techniques in primate models may help address these limitations.

### Conclusion

Evolutionary conservation and divergence across species are important to understanding brain functions in mammals. Our findings demonstrate that intrinsic heterogeneity of MCs across the septotemporal axis is conserved between mouse and monkey, despite notable differences in axonal projections between these two species. In the associational projections, temporal MCs project to the IML extent to the septotemporal axis, consistent across species, whereas septal MCs in monkey have distinct axonal projections depending on the septotemporal axis of the DG, displaying differential topological organization with mouse septal MCs. Moreover, in the contralateral projections, monkey lacks commissural projections along the septotemporal axis, contrasting with the presence of such projections in mice. Our study may provide clues to address the functional evolution of the hippocampal network for higher-order cognition.

## Synthesis

Reviewing Editor: Lynda Erskine, University of Aberdeen Decisions are customarily a result of the Reviewing Editor and the peer reviewers coming together and discussing their recommendations until a consensus is reached. When revisions are invited, a fact-based synthesis statement explaining their decision and outlining what is needed to prepare a revision will be listed below. The following reviewer(s) agreed to reveal their identity: NONE. Note: If this manuscript was transferred from JNeurosci and a decision was made to accept the manuscript without peer review, a brief statement to this effect will instead be what is listed below.

Thanks you for transferring your manuscript for consideration by eNeuro and for your careful responses to the reviewers' comments. I invited the original reviewers to evaluate the resubmission but neither accepted the invitation. Rather than send to new reviewers, I carefully evaluated the manuscript and the responses to the reviewers' comments and am satisfied that you have appropriately addressed all the points raised by the reviewers. There clearly is value in comparing mossy cells in the two species in a single study, albeit with some limitations due to the number of animals studies (which is clearly acknowledged in your revised manuscript). My decision is therefore to accept this manuscript for publication in eNeuro.
