# Supplementary figures and images for: Comparative Anatomy of the Dentate Mossy Cells in Nonhuman Primates: Their Spatial Distributions and Axonal Projections Compared With Mouse Mossy Cells
Source: eNeuro. 2024 May 10;11(5):ENEURO.0151-24.2024. doi: 10.1523/ENEURO.0151-24.2024 (PMC11151194; doi:10.1523/ENEURO.0151-24.2024)

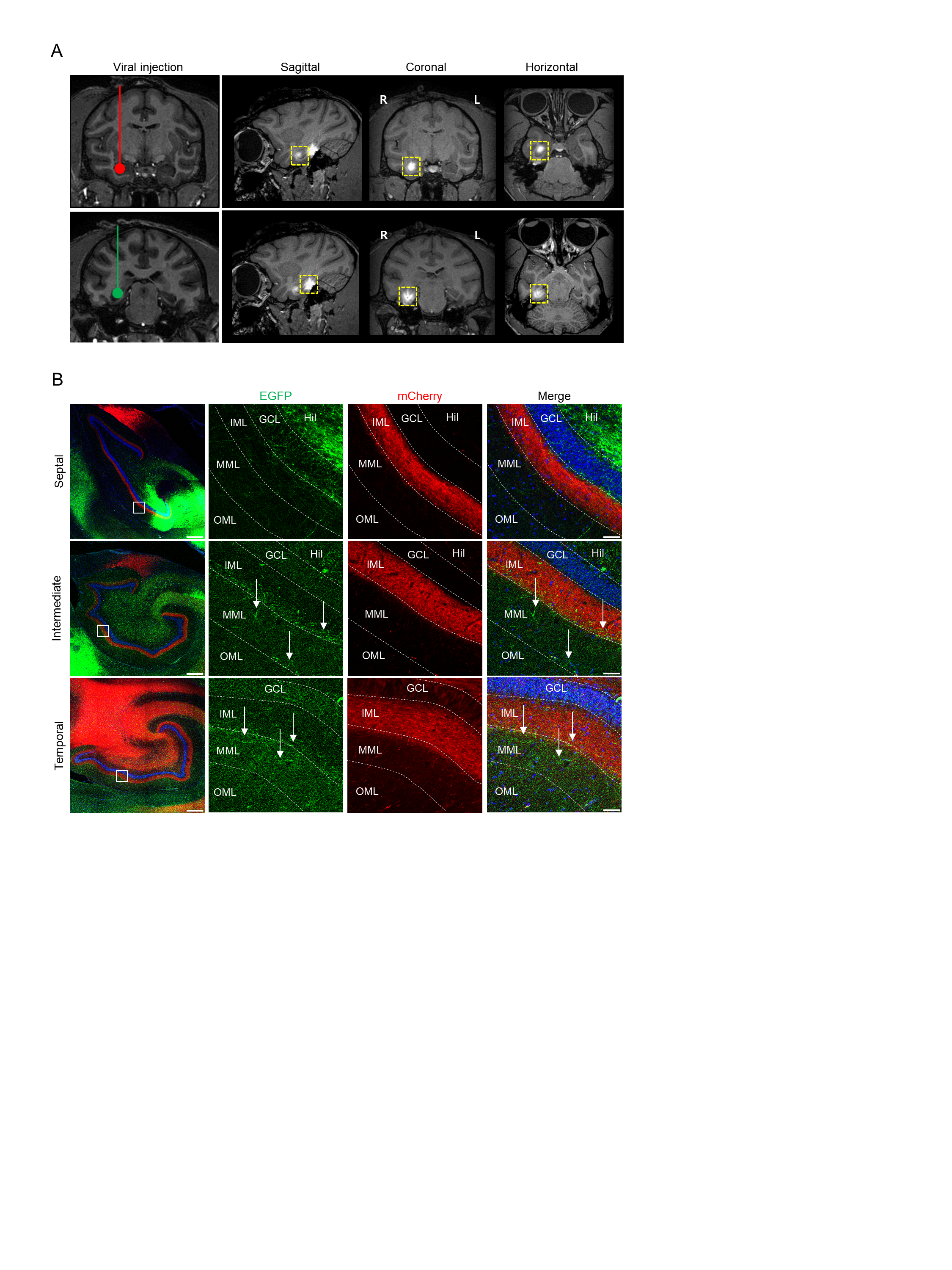

Supplement: Figure 5-1 — Septotemporal heterogeneity of MCs in their axonal projections in the molecular layers of the DG in another monkey subject. A, Schematics for viral injections in a monkey. The septal and temporal DG of a rhesus monkey were injected with AAVs expressing EGFP and mCherry under the CaMKIIa promoter, respectively. MRI imaging with multiple planes shows viral injection in a location-specific manner. An MRI contrast agent was co-infused with AAVs to visualize the injection site. B, Representative images showing axonal projections of septal and temporal MCs along the septotemporal axis of the DG in the monkey. Labeling of septal MC, Bregma: -20.50 mm, approximately 10% in the hilus; labeling of temporal MC, Bregma: -12.60 mm, approximately 80% in the hilus. The scale bars represent 500 μm for large-scale images and 50 μm for zoomed images. Distribution pattern of axonal fibers from septal (EGFP) and temporal (mCherry) MCs in each molecular layer of the monkey DG. Arrows indicate axonal fibers from septal (EGFP) at each molecular layer. Hil, hilus; GCL, granule cell layer; IML, inner molecular layer; MML, middle molecular layer; OML, outer molecular layer. Download Figure 5-1, TIF file. [file eneuro-11-ENEURO.0151-24.2024-s002.tif]
